# Genetic Dissection of Grain Yield of Maize and Yield-Related Traits Through Association Mapping and Genomic Prediction

**DOI:** 10.3389/fpls.2021.690059

**Published:** 2021-07-15

**Authors:** Juan Ma, Yanyong Cao

**Affiliations:** Institute of Cereal Crops, Henan Academy of Agricultural Sciences, Zhengzhou, China

**Keywords:** grain yield, genome-wide association study, trait-associated markers, prediction accuracy, fixed model

## Abstract

High yield is the primary objective of maize breeding. Genomic dissection of grain yield and yield-related traits contribute to understanding the yield formation and improving the yield of maize. In this study, two genome-wide association study (GWAS) methods and genomic prediction were made on an association panel of 309 inbred lines. GWAS analyses revealed 22 significant trait–marker associations for grain yield per plant (GYP) and yield-related traits. Genomic prediction analyses showed that reproducing kernel Hilbert space (RKHS) outperformed the other four models based on GWAS-derived markers for GYP, ear weight, kernel number per ear and row, ear length, and ear diameter, whereas genomic best linear unbiased prediction (GBLUP) showed a slight superiority over other modes in most subsets of the trait-associated marker (TAM) for thousand kernel weight and kernel row number. The prediction accuracy could be improved when significant single-nucleotide polymorphisms were fitted as the fixed effects. Integrating information on population structure into the fixed model did not improve the prediction performance. For GYP, the prediction accuracy of TAMs derived from fixed and random model Circulating Probability Unification (FarmCPU) was comparable to that of the compressed mixed linear model (CMLM). For yield-related traits, CMLM-derived markers provided better accuracies than FarmCPU-derived markers in most scenarios. Compared with all markers, TAMs could effectively improve the prediction accuracies for GYP and yield-related traits. For eight traits, moderate- and high-prediction accuracies were achieved using TAMs. Taken together, genomic prediction incorporating prior information detected by GWAS could be a promising strategy to improve the grain yield of maize.

## Introduction

Maize serves as an important cereal and forage crop and plays an important role in sustaining global food security. Improvement of grain yield is a major and longstanding breeding goal for maize. Kernel number per ear (KNE) and thousand kernel weight (HKW) are the major components of grain yield per plant (GYP). Kernel number per row (KNR) and kernel row number (KRN) are the important components of the KNE. Ear length (EL) and ear diameter (ED) affect GYP in different degrees. In general, compared to GYP, yield components and related traits are less affected by environments and have higher heritability, and therefore, can be directly used to facilitate the final yield of maize (Shi et al., 2017). Identifying loci associated with GYP and yield-related traits will contribute to understanding their basis and the correlations between them at a molecular level. In addition, the identification of important loci and genes involved will provide useful information for whole-genome selection of high-yield potential.

Using linkage mapping and genome-wide association study (GWAS), a large number of quantitative trait loci (QTLs) or single-nucleotide polymorphisms (SNPs) have been identified among different populations. For instance, under drought and heat environments, Millet et al. (2016) detected a large number of significant SNPs for the grain yield and the grain number using single-environment and multi-environment GWAS methods. Zhang et al. (2017) identified 23 QTLs and 25 significant SNPs for HKW, KRN, and KNR in recombinant inbred lines and an association panel of 240 maize inbred lines, and a stable locus (*PKS2*) influencing KRN, HKW, and kernel shapes was identified. Using an intermated B73 × Mo17 Syn10 doubled haploid population and a natural population, Zhang et al. (2020) detected 100 QTLs and 138 SNPs for GYP and yield-related traits and found that eight significant SNPs were co-located within intervals of seven QTLs. These studies enforce the complex of GYP and yield-related traits, which are governed by a mixture of many large-effect and small-effect genomic components.

Traditional marker-assisted selection (MAS) and marker-assisted recurrent selection (MARS) use only a few large-effect QTLs or markers, where efficient selections are made in maize breeding programs. Genomic selection (GS) uses whole genome-wide molecular markers to predict the breeding values of individuals. Therefore, it can capture both major and minor effect markers and is efficient for complex traits, especially for grain yield. GS has been shown to outperform MAS for grain yield and physiological traits in maize doubled haploid populations (Cerrudo et al., 2018), and for days to silking/anthesis and anthesis–silking interval in a nested association mapping population (Guo et al., 2021). Annual gain from GS outperformed that from MAS by 2-fold for winter wheat and approximately 3-fold for maize at a moderate accuracy (Heffner et al., 2010). Genetic gains of maize stover index and yield + stover index were 14–50% larger with GS than with MARS (Massman et al., 2013), which is consistent with the simulation results that GS produced up to 43% greater genetic gains than MARS for polygenic traits with low heritability (Bernardo and Yu, 2007). The primary advantages of GS over phenotypic selection are reflected in its low cost per cycle and the time for variety development. In maize advanced test-cross yield trials, GS reduced the cost by 32% over phenotype-based selection with similar selection gains (Beyene et al., 2019). With respect to cost reduction in maize breeding, breeders can test-cross half of all available lines, evaluate them in first-stage multi-environment trials, and then utilize the phenotypic data to predict the remaining half through GS (Crossa et al., 2017).

In GS, prediction models are established using prior phenotypic and marker data in a training population. The genomic estimated breeding value (GEBV) is predicted based on the marker effects estimated from the training population in a test population with genotypic data and no phenotypic data (Meuwissen et al., 2001). Many parametric methods such as GBLUP and Bayesian (Bayes) methods including Bayes A, Bayes B, Bayes C, and Bayes least absolute shrinkage and selection operator, semi-parametric models such as RKHS, and nonparametric methods have been developed to fit marker effects and predict phenotypes (Meuwissen et al., 2001; Gianola et al., 2006, 2011; Parmley et al., 2019; Sun et al., 2020). Multivariate models were developed to simultaneously consider information from multi-environment trials or multi-trait data (Burgueño et al., 2012; Montesinos-López et al., 2016; Schulthess et al., 2018). Previous studies showed that no single GS model had better performance compared with other models in all cases due to different backgrounds of training and testing populations, different traits, and different experimental designs (Pérez-Rodríguez et al., 2012; Ali et al., 2020). In maize, practical applications of GS have been widely demonstrated in many aspects including inbred line prediction (Zhao et al., 2012; Liu et al., 2019), hybrid performance prediction (Guo et al., 2019; Schrag et al., 2019; Li et al., 2020), and combining ability prediction (Riedelsheimer et al., 2012). These findings demonstrate the potential of GS helping in the selection of elite parents and hybrid combinations.

Both GWAS and GS use the same input datasets, including a phenotype dataset and a genotype dataset; thus, only additional analyses are required (Spindel et al., 2016). Several studies have discussed the advantages of combining GWAS and GS models that incorporate trait-associated markers (TAMs) detected by GWAS as random or fixed effects in GS models (Spindel et al., 2016; Bian and Holland, 2017; Herter et al., 2019; Liu et al., 2019; Rice and Lipka, 2019). However, the effects of TAM derived from different GWAS methods on prediction accuracy have rarely been reported. In this study, an association panel of 309 inbred lines was genotyped with 58,129 markers using genotyping-by-sequencing (GBS), and the performance of GYP, ear weight (EW), HKW, KNE, KNR, KRN, EL, and ED was evaluated in multi-environment trials. The main objectives of this study were to (1) identify significant SNPs for eight traits using two GWAS methods, (2) compare the prediction accuracies of different GS models, (3) investigate the prediction accuracy by treating significant SNPs and population structure as the fixed effects, and (4) evaluate the effects of TAMs derived from different GWAS methods on prediction accuracy.

## Materials and Methods

### Plant Materials and Trial Designs

The panel consisted of 16 new selected inbred lines, 128 core germplasms of China, and 165 expired U.S. plant variety protection inbred lines, as previously reported (Ma et al., 2021). The panel was evaluated at four sites: Dancheng (33.646° N, 115.257° E), Yuanyang (35.012° N, 113.704° E), Yucheng (34.411° N, 116.274° E), and Sanya (18.381° N, 109.183° E) in 2017, and at one site (Yuanyang) in 2019. The field trial had a randomized complete block design with three replicates per genotype and environment. Entries were planted in two-row plots that were 3.75 m in length, 0.60 m spacing between rows, and 0.33 m spacing between plants.

### Phenotyping and Analyses

Grain yield per plant, EW, HKW, KRN, KNR, EL, and ED were measured manually in three ears with good self-pollination for each genotype. KNE was calculated from KRN and KNR. Heritability at the per mean level and multi-environment ANOVA were calculated using QTL IciMapping v4.0 software (Meng et al., 2015). Pearson's correlation coefficient was calculated using the R package Performance Analytics. Best linear unbiased estimate (BLUE) values of each trait were calculated using QTL IciMapping v4.0 and were used as phenotypes for GWAS and GS analyses.

### Association Mapping Analysis

The GBS genotypic data of the panel have been described in a previous study (Ma et al., 2021). Markers with minor allele frequencies (MAF) less than 5%, missing rates greater than 10%, and heterozygous rates greater than 10% were removed. Finally, 58,129 SNPs were adopted for GWAS. The kinship matrix was calculated using the Centered_IBS method in TASSEL v5.2.60 (Bradbury et al., 2007). The subgroups (*K*) were estimated using the Bayesian Markov chain Monte Carlo method in Structure v2.3.4 (Pritchard et al., 2000). The Q matrix of two subgroups (*K* = 2) was used to control the population structure as previously described (Ma et al., 2021). To reduce false associations, a single-locus method, namely, compressed mixed linear model (CMLM) (Zhang et al., 2010), and one multi-locus method, namely, fixed and random model Circulating Probability Unification (FarmCPU) (Liu et al., 2016), were carried out using the GAPIT package (Lipka et al., 2012). The Q and K matrices were incorporated into both GWAS methods. A multiple testing correction is not required in multi-locus methods because all loci are estimated and tested simultaneously (Zhang et al., 2019b). Therefore, a less stringent *p*-value threshold of 1/58,129 = 1.72E−05 was used to identify significant SNPs in the two GWAS methods. Other parameters were set default based on the GAPIT manual. Linear regression was used to calculate the phenotypic variation explained (PVE) of FarmCPU, whereas the PVE of CMLM was calculated using GAPIT. Candidate genes were scanned from 50 kb upstream to downstream of each significant locus using ANNOVAR (Wang et al., 2010).

### Genomic Prediction

The prediction was done using GBLUP, Bayes A, Bayes B, Bayes C, and RKHS. Kernel averaging was used in the RKHS, and bandwidth parameters were set at 1/5M, 1/M, and 5/M, where M is the median squared Euclidean distance. Seven subset sizes of TAMs, that is, 100, 500, 1,000, 5,000, 10,000, 20,000, and 40,000 were selected according to the ranks of –log_10_(*p* value) calculated by FarmCPU and CMLM based on BLUE values. The prediction accuracy of seven subsets was compared to that of all markers (58,129). For the eight traits, TAMs were all treated as the random effects (random model) in all GS models. For traits where significant SNPs (*p* <1.72E−05) were detected, the significant SNPs were treated as the fixed effects and other remaining markers were treated as the random effects (fixed model). In the fixed model, one Q matrix (Q1) calculated using Structure was added into GBLUP and RKHS models as the fixed effects to evaluate the impact of population structure on the prediction accuracy. In addition, significant SNPs were all fitted as the random effects in RKHS to evaluate their potential application.

Randomized imputation was adopted for missing makers, according to the known genotype frequency. For each marker, individuals were coded as 2 (homozygous minor allele), 0 (homozygous major allele), and 1 (heterozygous). Recoding and imputation were carried out using the R software. Five GS models, TAMs, fixed model, random model, and fixed effects of Q matrix were performed using the R package, BGLR (Pérez and de los Campos, 2014). For all models, the length of the Gibbs chain was 12,000 iterations, with the first 3,000 samples discarded as burn-in. A 5-fold cross-validation scheme with 100 replicates was used to divide the association panel into training and testing sets. The mean correlation coefficient between GEBVs and BLUE values in the testing sets was used to estimate the accuracies of different GS models and different SNP densities.

## Results

### Phenotypic Descriptions and Correlations

Descriptive statistics revealed that extensive phenotypic variations were observed in GYP and seven yield-related traits in the panel under different environments (Supplementary Table 1). The heritability for eight traits ranged from 0.59 (EW and KRN) to 0.77 (EL) (Supplementary Table 2). Significant and positive pairwise correlations were observed between different traits. GYP had high correlations with EW, KNE, KNR, and ED, moderate correlations with KRN and EL, and low correlations with HKW (Supplementary Figure 1). ANOVA across environments showed that the effects of genotype, environment, and genotype × environment interactions were significant (*p* < 0.001) for all traits (Supplementary Table 2). This showed that the association panel was highly affected by environments. Therefore, the BLUE values were used for GWAS and GS analyses.

### Significant Trait Marker Associations and Their Prediction Accuracies

In total, 58,129 high-quality SNPs were used to perform GWAS for eight traits using BLUE values. FarmCPU and CMLM were used to control false associations for all traits. A total of 22 significant SNPs were identified with a *p*-value threshold of 1.72E−05, and the average PVE of all significant signals was 4.20% (Table 1). FarmCPU detected 17 association signals, which was higher than CMLM (7) (Table 1). One significant SNP each was found for GYP, EW, and HKW. Eight, eight, and four significant SNPs were detected for KRN, ED, and EL, respectively. One pleiotropic SNP (S3_62750920) was found between EW and ED. A SNP for ED, namely, S7_174915679, was detected using the two GWAS methods. The prediction accuracy of the significant SNPs was ranged from 0.26 to 0.45 using RKHS (Supplementary Figure 2).

**Table 1:** Significant SNPs and candidate genes for grain yield and yield-related traits using two GWAS methods.

SNP name^*^	**Trait^§^**	***p* value**	**PVE^†^**	**Method^#^**	**Candidate gene**
S3_53872814	GYP	1.68E−05	5.92	FarmCPU	Zm00001d040612
S3_62750920	EW	1.02E−05	5.98	FarmCPU	Zm00001d040748, Zm00001d040751
S1_47210783	HKW	1.56E−05	6.16	CMLM	Zm00001d028812
S1_10685412	KRN	1.43E−05	1.99	FarmCPU	Zm00001d027671
S1_179199207	KRN	3.38E−06	4.80	FarmCPU	Zm00001d031137, Zm00001d031138
S3_134708533	KRN	2.45E−06	1.44	FarmCPU	Zm00001d041715, Zm00001d041716
S4_135839291	KRN	2.79E−06	1.32	FarmCPU	Zm00001d050992
S4_234082607	KRN	1.54E−07	2.29	FarmCPU	Zm00001d053559
S4_86484873	KRN	1.08E−07	1.74	FarmCPU	Zm00001d050406, Zm00001d050409
S7_105588532	KRN	5.13E−08	7.38	FarmCPU	Zm00001d020310, Zm00001d020311
S8_145121832	KRN	2.46E−06	0	FarmCPU	Zm00001d011266
S1_69620597	EL	5.84E−07	1.35	FarmCPU	Zm00001d029416
S3_174651102	EL	2.11E−08	7.21	FarmCPU	Zm00001d042631, Zm00001d042632
S4_117775505	EL	7.78E−06	0.70	FarmCPU	Zm00001d050712, Zm00001d050714
S4_174433366	EL	4.36E−06	4.80	FarmCPU	Zm00001d051912
S1_233432714	ED	7.53E−06	10.26	FarmCPU	Zm00001d032659, Zm00001d032661
S2_118387989	ED	1.47E−05	5.43	CMLM	Zm00001d004568, Zm00001d004571
S2_118390724	ED	1.59E−05	5.39	CMLM	Zm00001d004568, Zm00001d004571
S2_118625688	ED	1.46E−05	5.43	CMLM	Zm00001d004572, Zm00001d004573
S2_118744667	ED	1.21E−05	5.54	CMLM	Zm00001d004573, Zm00001d004574
S3_62750920	ED	1.01E−05	5.64	CMLM	Zm00001d040748, Zm00001d040751
S7_13345176	ED	4.01E−06	3.77	FarmCPU	Zm00001d019027, Zm00001d019028
S7_174915679	ED	1.22E−05	5.54	CMLM	Zm00001d022310
S7_174915679	ED	3.94E−06	0.76	FarmCPU	Zm00001d022310

* *Numbers before and after “_” represent chromosome and position, respectively*.

§ *GYP, EW, HKW, KRN, EL, and ED are abbreviations of grain yield per plant, ear weight, thousand kernel weight, kernel row number, ear length, and ear diameter, respectively*.

† *PVE, phenotypic variation explained*.

# *CMLM, compressed mixed linear model; FarmCPU, fixed and random model Circulating Probability Unification*.

### Prediction Accuracy of Different Prediction Models

Five GS models were evaluated using seven subsets of TAMs derived from FarmCPU and CMLM. The prediction accuracies ranged from 0.10 to 0.84 and differed among prediction models and traits. Regardless of the marker effects, the prediction accuracy of RKHS using TAMs was the highest, followed by GBLUP, and Bayes B was the least for GYP, EW, and KNE (Tables 2 and 3, Supplementary Table 3). The prediction accuracies of the RKHS exceeded those of the other models by 3.85–68% for GYP and by 1.52–33.33% for KNE (Table 2, Supplementary Table 3). For EW, the percentage increase in accuracy of RKHS over the other four models using CMLM-derived TAMs ranged from 1.85 to 64%, whereas that of RKHS over the other models using FarmCPU-derived TAMs was large, with the percentage increase ranging from 26.09 to 210% (Table 3). Slight increases in the prediction accuracies of RKHS over the other models were also demonstrated in most subsets for KNR, EL, and ED (Supplementary Tables 4–6). For HKW, GBLUP was slightly superior to RKHS, Bayes A, Bayes B, and Bayes C (Table 4). In most of the marker sets, a small advantage of GBLUP over other models was also observed in KRN (Table 5).

**Table 2:** Prediction accuracy of random model, fixed model, and population structure model based on trait-associated markers in five prediction models for grain yield per plant.

Model^*^	**Scenario^**§**^**	**Prediction accuracy**^****#****^							
		**100^**†**^**	**500**	**1,000**	**5,000**	**10,000**	**20,000**	**40,000**	**58,129**
Bayes A	CMLM-RAN	0.51 (0.08)	0.56 (0.07)	0.56 (0.08)	0.56 (0.08)	0.53 (0.08)	0.47 (0.09)	0.29 (0.11)	0.09 (0.12)
	FarmCPU-RAN	0.51 (0.08)	0.56 (0.07)	0.56 (0.07)	0.56 (0.08)	0.53 (0.08)	0.46 (0.09)	0.29 (0.11)	
	FarmCPU-FIX	0.52 (0.08)	0.56 (0.08)	0.56 (0.08)	0.57 (0.08)	0.54 (0.09)	0.47 (0.10)	0.33 (0.11)	
Bayes B	CMLM-RAN	0.48 (0.09)	0.53 (0.08)	0.53 (0.08)	0.54 (0.08)	0.51 (0.09)	0.44 (0.09)	0.26 (0.11)	0.08 (0.12)
	FarmCPU-RAN	0.48 (0.09)	0.54 (0.08)	0.53 (0.08)	0.54 (0.08)	0.51 (0.09)	0.44 (0.09)	0.25 (0.11)	
	FarmCPU-FIX	0.49 (0.09)	0.54 (0.08)	0.54 (0.08)	0.56 (0.08)	0.53 (0.09)	0.45 (0.10)	0.32 (0.12)	
Bayes C	CMLM-RAN	0.50 (0.09)	0.55 (0.07)	0.55 (0.08)	0.56 (0.08)	0.53 (0.08)	0.46 (0.09)	0.28 (0.12)	0.09 (0.12)
	FarmCPU-RAN	0.50 (0.09)	0.55 (0.07)	0.55 (0.07)	0.56 (0.08)	0.53 (0.08)	0.46 (0.09)	0.28 (0.11)	
	FarmCPU-FIX	0.51 (0.09)	0.56 (0.08)	0.57 (0.08)	0.57 (0.08)	0.53 (0.09)	0.46 (0.10)	0.33 (0.11)	
GBLUP	CMLM-RAN	0.52 (0.08)	0.57 (0.07)	0.57 (0.08)	0.59 (0.08)	0.56 (0.09)	0.49 (0.09)	0.30 (0.11)	0.10 (0.12)
	FarmCPU-RAN	0.52 (0.08)	0.57 (0.07)	0.57 (0.07)	0.59 (0.08)	0.55 (0.09)	0.48 (0.09)	0.30 (0.11)	
	FarmCPU-FIX	0.52 (0.08)	0.57 (0.07)	0.57 (0.08)	0.57 (0.08)	0.53 (0.09)	0.46 (0.10)	0.33 (0.12)	
	FarmCPU-FIX-PS	0.52 (0.08)	0.57 (0.08)	0.57 (0.08)	0.57 (0.08)	0.53 (0.09)	0.46 (0.10)	0.32 (0.12)	
RKHS	CMLM-RAN	0.54 (0.09)	0.62 (0.07)	0.61 (0.08)	0.62 (0.08)	0.59 (0.09)	0.54 (0.10)	0.42 (0.12)	0.32 (0.14)
	FarmCPU-RAN	0.54 (0.09)	0.62 (0.07)	0.61 (0.08)	0.62 (0.08)	0.59 (0.09)	0.54 (0.10)	0.42 (0.12)	
	FarmCPU-FIX	0.54 (0.08)	0.61 (0.08)	0.61 (0.08)	0.61 (0.08)	0.57 (0.09)	0.52 (0.10)	0.42 (0.11)	
	FarmCPU-FIX-PS	0.54 (0.09)	0.61 (0.08)	0.61 (0.08)	0.61 (0.08)	0.57 (0.09)	0.52 (0.10)	0.42 (0.11)	

*GBLUP, genomic best linear unbiased prediction; RKHS, reproducing kernel Hilbert space.

*^§^CMLM-RAN and FarmCPU-RAN, traits-associated markers from compressed mixed linear model (CMLM) and fixed and random model Circulating Probability Unification (FarmCPU) are treated as random effects; FarmCPU-FIX, significant SNPs (p <1.72E−05) are treated as the fixed effects and other remaining markers are treated as the random effects (fixed model); FarmCPU-FIX-PS, the Q matrix is treated as fixed effect in the fixed model*.

*^†^100–40,000, the number of trait-associated markers*.

*^#^Prediction accuracy is represented by mean and standard deviation in brackets*.

**Table 3:** Prediction accuracy of random model, fixed model, and population structure model based on trait-associated markers in five prediction models for ear weight.

Model^*^	**Scenario^**§**^**	**Prediction accuracy**^****#****^							
		**100^**†**^**	**500**	**1,000**	**5,000**	**10,000**	**20,000**	**40,000**	**58,129**
Bayes A	CMLM-RAN	0.54 (0.09)	0.58 (0.08)	0.57 (0.08)	0.55 (0.09)	0.50 (0.09)	0.44 (0.10)	0.27 (0.12)	
	FarmCPU-RAN	0.20 (0.11)	0.12 (0.11)	0.15 (0.10)	0.19 (0.11)	0.18 (0.11)	0.14 (0.12)	0.10 (0.12)	0.09 (0.12)
	FarmCPU-FIX	0.19 (0.12)	0.14 (0.11)	0.18 (0.11)	0.20 (0.11)	0.19 (0.11)	0.16 (0.11)	0.12 (0.12)	
Bayes B	CMLM-RAN	0.51 (0.09)	0.55 (0.08)	0.54 (0.08)	0.53 (0.09)	0.48 (0.09)	0.41 (0.10)	0.25 (0.12)	
	FarmCPU-RAN	0.16 (0.11)	0.13 (0.11)	0.16 (0.11)	0.18 (0.11)	0.17 (0.12)	0.14 (0.12)	0.11 (0.12)	0.09 (0.12)
	FarmCPU-FIX	0.15 (0.11)	0.13 (0.11)	0.18 (0.11)	0.20 (0.12)	0.19 (0.12)	0.16 (0.12)	0.12 (0.12)	
Bayes C	CMLM-RAN	0.53 (0.09)	0.57 (0.08)	0.57 (0.08)	0.55 (0.09)	0.50 (0.09)	0.43 (0.10)	0.27 (0.12)	
	FarmCPU-RAN	0.23 (0.11)	0.17 (0.11)	0.18 (0.11)	0.19 (0.11)	0.18 (0.11)	0.14 (0.12)	0.11 (0.12)	0.09 (0.12)
	FarmCPU-FIX	0.20 (0.12)	0.16 (0.11)	0.20 (0.11)	0.20 (0.11)	0.19 (0.11)	0.15 (0.12)	0.12 (0.12)	
GBLUP	CMLM-RAN	0.54 (0.09)	0.59 (0.08)	0.58 (0.08)	0.58 (0.09)	0.53 (0.09)	0.46 (0.10)	0.29 (0.12)	0.12 (0.12)
	FarmCPU-RAN	0.20 (0.12)	0.19 (0.11)	0.23 (0.11)	0.22 (0.11)	0.20 (0.11)	0.16 (0.12)	0.12 (0.12)	
	FarmCPU-FIX	0.18 (0.12)	0.17 (0.11)	0.20 (0.11)	0.20 (0.11)	0.19 (0.12)	0.16 (0.12)	0.12 (0.12)	
	FarmCPU-FIX-PS	0.17 (0.12)	0.16 (0.11)	0.19 (0.11)	0.20 (0.11)	0.19 (0.12)	0.15 (0.12)	0.12 (0.12)	
RKHS	CMLM-RAN	0.55 (0.09)	0.62 (0.08)	0.61 (0.08)	0.61 (0.09)	0.57 (0.09)	0.52 (0.11)	0.41 (0.13)	0.31 (0.14)
	FarmCPU-RAN	0.29 (0.13)	0.33 (0.13)	0.37 (0.12)	0.37 (0.13)	0.34 (0.13)	0.32 (0.14)	0.31 (0.14)	
	FarmCPU-FIX	0.28 (0.14)	0.28 (0.13)	0.31 (0.13)	0.37 (0.13)	0.36 (0.13)	0.33 (0.14)	0.31 (0.14)	
	FarmCPU-FIX-PS	0.27 (0.13)	0.27 (0.13)	0.31 (0.13)	0.36 (0.13)	0.36 (0.13)	0.33 (0.14)	0.31 (0.14)	

*GBLUP, genomic best linear unbiased prediction; RKHS, reproducing kernel Hilbert space.

*^§^CMLM-RAN and FarmCPU-RAN, traits-associated markers from compressed mixed linear model (CMLM) and fixed and random model Circulating Probability Unification (FarmCPU) are treated as random effects; FarmCPU-FIX, significant SNPs (p <1.72E−05) are treated as the fixed effects and other remaining markers are treated as the random effects (fixed model); FarmCPU-FIX-PS, the Q matrix is treated as the fixed effect in the fixed model*.

*^†^100–40,000, the number of trait-associated markers*.

*^#^Prediction accuracy is represented by mean and standard deviation in brackets*.

**Table 4:** Prediction accuracy of random model, fixed model, and population structure model based on trait-associated markers in five prediction models for thousand kernel weight.

Model^*^	**Scenario^**§**^**	**Prediction accuracy**^****#****^							
		**100^**†**^**	**500**	**1,000**	**5,000**	**10,000**	**20,000**	**40,000**	**58,129**
Bayes A	CMLM-RAN	0.65 (0.06)	0.72 (0.06)	0.72 (0.05)	0.70 (0.05)	0.67 (0.06)	0.60 (0.07)	0.41 (0.08)	
	FarmCPU-RAN	0.55 (0.08)	0.58 (0.07)	0.59 (0.07)	0.58 (0.07)	0.54 (0.07)	0.48 (0.08)	0.35 (0.08)	0.20 (0.09)
	CMLM-FIX	0.66 (0.06)	0.72 (0.05)	0.73 (0.05)	0.71 (0.05)	0.67 (0.06)	0.60 (0.07)	0.42 (0.09)	
Bayes B	CMLM-RAN	0.63 (0.06)	0.70 (0.06)	0.71 (0.05)	0.68 (0.06)	0.64 (0.06)	0.57 (0.07)	0.39 (0.08)	0.21 (0.09)
	FarmCPU-RAN	0.53 (0.08)	0.56 (0.07)	0.58 (0.08)	0.56 (0.07)	0.52 (0.07)	0.46 (0.08)	0.34 (0.09)	
	CMLM-FIX	0.64 (0.06)	0.71 (0.05)	0.71 (0.05)	0.69 (0.06)	0.66 (0.06)	0.58 (0.07)	0.41 (0.09)	
Bayes C	CMLM-RAN	0.65 (0.06)	0.72 (0.06)	0.72 (0.05)	0.70 (0.05)	0.67 (0.06)	0.60 (0.07)	0.41 (0.08)	0.20 (0.09)
	FarmCPU-RAN	0.55 (0.08)	0.57 (0.07)	0.59 (0.07)	0.58 (0.07)	0.54 (0.07)	0.48 (0.08)	0.35 (0.08)	
	CMLM-FIX	0.66 (0.06)	0.72 (0.05)	0.73 (0.05)	0.71 (0.05)	0.67 (0.06)	0.60 (0.07)	0.42 (0.09)	
GBLUP	CMLM-RAN	0.67 (0.06)	0.73 (0.05)	0.73 (0.05)	0.71 (0.05)	0.68 (0.06)	0.60 (0.07)	0.40 (0.08)	0.20 (0.09)
	FarmCPU-RAN	0.56 (0.08)	0.60 (0.07)	0.60 (0.07)	0.58 (0.07)	0.54 (0.07)	0.48 (0.08)	0.34 (0.08)	
	CMLM-FIX	0.67 (0.06)	0.73 (0.05)	0.73 (0.05)	0.71 (0.05)	0.67 (0.06)	0.60 (0.07)	0.42 (0.09)	
	CMLM-FIX-PS	0.66 (0.06)	0.72 (0.05)	0.72 (0.05)	0.69 (0.05)	0.64 (0.06)	0.55 (0.07)	0.39 (0.09)	
RKHS	CMLM-RAN	0.66 (0.06)	0.72 (0.05)	0.72 (0.05)	0.69 (0.06)	0.65 (0.06)	0.56 (0.07)	0.37 (0.08)	0.24 (0.08)
	FarmCPU-RAN	0.54 (0.08)	0.58 (0.07)	0.58 (0.07)	0.55 (0.07)	0.51 (0.07)	0.45 (0.08)	0.33 (0.08)	
	CMLM-FIX	0.66 (0.06)	0.72 (0.05)	0.72 (0.05)	0.69 (0.06)	0.64 (0.06)	0.55 (0.07)	0.39 (0.09)	
	CMLM-FIX-PS	0.66 (0.06)	0.72 (0.05)	0.72 (0.05)	0.69 (0.05)	0.64 (0.06)	0.55 (0.07)	0.39 (0.09)	

*GBLUP, genomic best linear unbiased prediction; RKHS, reproducing kernel Hilbert space.

*^§^CMLM-RAN and FarmCPU-RAN, traits-associated markers from compressed mixed linear model (CMLM) and fixed and random model Circulating Probability Unification (FarmCPU) are treated as the random effects; CMLM-FIX, significant SNPs (p <1.72E−05) are treated as the fixed effects and other remaining markers are treated as the random effects (fixed model); CMLM-FIX-PS, the Q matrix is treated as the fixed effect in the fixed model*.

*^†^100–40,000, the number of trait-associated markers*.

*^#^Prediction accuracy is represented by mean and standard deviation in brackets*.

**Table 5:** Prediction accuracy of random model, fixed model, and population structure model based on trait-associated markers in five prediction models for kernel row number.

Model^*^	**Scenario^**§**^**	**Prediction accuracy**^****#****^							
		**100^**†**^**	**500**	**1,000**	**5,000**	**10,000**	**20,000**	**40,000**	**58,129**
Bayes A	CMLM-RAN	0.71 (0.06)	0.77 (0.05)	0.79 (0.05)	0.78 (0.05)	0.76 (0.06)	0.69 (0.07)	0.52 (0.10)	0.34 (0.12)
	FarmCPU-RAN	0.70 (0.05)	0.68 (0.07)	0.64 (0.07)	0.57 (0.10)	0.55 (0.09)	0.49 (0.10)	0.41 (0.11)	
	FarmCPU-FIX	0.70 (0.05)	0.73 (0.06)	0.73 (0.08)	0.69 (0.07)	0.67 (0.07)	0.63 (0.08)	0.57 (0.09)	
Bayes B	CMLM-RAN	0.69 (0.07)	0.75 (0.05)	0.78 (0.05)	0.76 (0.06)	0.74 (0.06)	0.66 (0.08)	0.50 (0.10)	0.35 (0.12)
	FarmCPU-RAN	0.68 (0.05)	0.69 (0.06)	0.66 (0.07)	0.56 (0.09)	0.53 (0.10)	0.47 (0.11)	0.40 (0.11)	
	FarmCPU-FIX	0.69 (0.06)	0.73 (0.06)	0.73 (0.06)	0.68 (0.07)	0.66 (0.07)	0.62 (0.08)	0.56 (0.09)	
Bayes C	CMLM-RAN	0.70 (0.06)	0.77 (0.05)	0.79 (0.05)	0.78 (0.05)	0.76 (0.06)	0.69 (0.07)	0.52 (0.10)	0.35 (0.11)
	FarmCPU-RAN	0.69 (0.05)	0.69 (0.06)	0.64 (0.07)	0.56 (0.10)	0.55 (0.09)	0.48 (0.10)	0.41 (0.11)	
	FarmCPU-FIX	0.70 (0.05)	0.74 (0.06)	0.73 (0.06)	0.69 (0.07)	0.67 (0.07)	0.63 (0.08)	0.57 (0.09)	
GBLUP	CMLM-RAN	0.72 (0.06)	0.77 (0.05)	0.80 (0.05)	0.79 (0.05)	0.76 (0.06)	0.70 (0.07)	0.53 (0.10)	0.36 (0.12)
	FarmCPU-RAN	0.70 (0.05)	0.67 (0.07)	0.63 (0.08)	0.56 (0.10)	0.56 (0.10)	0.50 (0.11)	0.42 (0.11)	
	FarmCPU-FIX	0.70 (0.05)	0.73 (0.06)	0.73 (0.06)	0.69 (0.07)	0.67 (0.07)	0.63 (0.08)	0.57 (0.09)	
	FarmCPU-FIX-PS	0.71 (0.05)	0.73 (0.06)	0.73 (0.06)	0.69 (0.07)	0.67 (0.07)	0.63 (0.08)	0.57 (0.09)	
RKHS	CMLM-RAN	0.70 (0.06)	0.77 (0.05)	0.79 (0.05)	0.77 (0.06)	0.75 (0.06)	0.67 (0.07)	0.51 (0.09)	0.39 (0.10)
	FarmCPU-RAN	0.70 (0.05)	0.65 (0.07)	0.62 (0.08)	0.56 (0.09)	0.54 (0.09)	0.49 (0.10)	0.43 (0.10)	
	FarmCPU-FIX	0.71 (0.05)	0.72 (0.06)	0.72 (0.06)	0.67 (0.07)	0.65 (0.08)	0.61 (0.08)	0.56 (0.09)	
	FarmCPU-FIX-PS	0.71 (0.05)	0.72 (0.06)	0.72 (0.06)	0.67 (0.07)	0.65 (0.08)	0.61 (0.08)	0.56 (0.09)	

*GBLUP, genomic best linear unbiased prediction; RKHS, reproducing kernel Hilbert space.

*^§^CMLM-RAN and FarmCPU-RAN, traits-associated markers from compressed mixed linear model (CMLM) and fixed and random model Circulating Probability Unification (FarmCPU) are treated as the random effects; FarmCPU-FIX, significant SNPs (p <1.72E−05) are treated as the fixed effects and other remaining markers are treated as the random effects (fixed model); FarmCPU-FIX-PS, the Q matrix is treated as the fixed effect in the fixed model*.

*^†^100–40,000, the number of trait-associated markers*.

*^#^Prediction accuracy is represented by mean and standard deviation in brackets*.

### Impact of Using Significant SNPs and Population Structure as Fixed Effects on Prediction Accuracy

The prediction accuracies of using significant SNPs and population structure as the fixed effects were evaluated in traits where significant SNPs were detected. In most of the TAM subsets, using 4–8 significant SNPs as the fixed effects improved the prediction accuracy by 1.43–40% and 1.37–22.41% for KRN and EL, respectively, when compared with the random model in all five models (Table 5, Supplementary Table 5). For GYP, EW, and HKW, the prediction accuracy did not change (or slightly decreased) when treating one significant SNP as a fixed effect compared to fitting all markers as the random effects in GBLUP and RKHS. However, the accuracy of the fixed model slightly increased or was similar to that of the random model in the three Bayes prediction models. For ED, the fixed model based on FarmCPU-derived markers improved the accuracy by 1.35−16%, whereas that of CMLM-derived markers had similar prediction performance as the random model in most cases. In general, the prediction accuracy could be improved when significant SNPs were fitted as the fixed effects.

To evaluate the effect of population structure on prediction accuracies, the Q matrix calculated using Structure was incorporated into the fixed model in GBLUP and RKHS. For GYP, EW, and KRN, the accuracy did not change when the Q matrix was included as a fixed effect in most cases of RKHS and GBLUP (Tables 2, 3, 5). For HKW, the population structure had no effect on accuracies in RKHS, whereas the accuracy decreased by 0.01–0.05 when the Q matrix was used in the GBLUP model. For EL, the accuracy reduced by 0.02–0.07 at 500–20,000 TAMs when population structure was added into the GBLUP fixed model. For ED, the accuracy improved by 0.02 at 100 and 40,000 CMLM-derived TAMs and decreased by 0.05 at 40,000 FarmCPU-derived TAMs when the Q matrix was added in GBLUP, and the accuracy was same or slightly decreased in the remaining scenarios.

### Effect of Different GWAS Methods on Prediction Accuracy

For GYP, the prediction accuracies of TAMs derived from CMLM and FarmCPU were compared in the five models (Table 2), regardless of the random or fixed models. For EL and ED, the prediction accuracy of 100 TAMs by FarmCPU was 2.74–5.97% higher than that by CMLM in the five models. For the other subsets, the prediction accuracies of CMLM-derived markers were 8.22–42.11% higher than those of FarmCPU-derived markers in EL and ED (Supplementary Tables 5, 6). For the other five traits, the prediction accuracies of CMLM-TAMs were consistently superior to those of FarmCPU-TAMs across all subsets in the five models. In particular, the increase in prediction accuracies for CMLM-TAMs over FarmCPU-TAMs was large in EW, with the percentage increase ranging from 32.26 to 383.33% across all scenarios (Table 3). With respect to TAMs, moderate and high prediction accuracies were achieved in five prediction models for the eight traits. The optimum number of TAMs for prediction differed greatly among the eight traits, two GWAS methods, and five GS models. These results indicate that it is necessary to determine the optimum SNP information that can represent sufficient variations to achieve high prediction accuracies for each trait before their application in GS breeding. Compared to all SNPs, higher prediction accuracies were achieved using TAMs in most scenarios. This indicates that TAMs could effectively improve the prediction accuracies of GYP and yield-related traits.

## Discussion

Genomic selection is a promising breeding method with the aim of accelerating the speed and efficiency of breeding processes. In contrast, GWAS is used to identify QTLs or genes that underlie important traits for breeding. They seek to model the different aspects of the genetic architecture of traits and have complementary advantages (Bian and Holland, 2017). Previous studies have shown the effectiveness of the GS method using important loci for target traits identified by GWAS (Bian and Holland, 2017; Liu et al., 2019; Rice and Lipka, 2019). In this study, we demonstrated the potential of incorporating prior information for grain yield and seven yield-related traits explored by GWAS into GS in a maize association panel.

Prediction models are the major factors that affect the prediction accuracy of different traits. In this study, GBLUP, Bayes A, Bayes B, Bayes C, and RKHS were adopted to compare the prediction accuracies of eight traits based on GWAS-derived markers. The advantage of RKHS over the other four models was demonstrated using GYP, EW, KNE, KNR, EL, and ED in most TAM subsets, which was in line with many studies on maize, wheat, barley, and *Arabidopsis thaliana* (González-Camacho et al., 2012; Heslot et al., 2012; Pérez-Rodríguez et al., 2012; Liu et al., 2018; Li et al., 2020). RKHS, as one of the semiparametric methods, does not need to make most of the assumptions on the relationship between phenotype and genotype as do parametric models and was found to have the potential for capturing the total genetic effects from real data (Gianola et al., 2006; Gianola and van Kaam, 2008). The inferior performance of the RKHS over other models has also been reported in maize kernel oil traits (Hao et al., 2019) and cotton fiber quality traits (Islam et al., 2020). In this study, GBLUP showed a slight advantage over RKHS and the other models using TAMs for HKW and KRN. If additivity has a major effect, RKHS produces a similar performance as other methods, whereas if non-additive effects are present, it has a better prediction accuracy (Morota and Gianola, 2014). Although no single model was consistently performing better in all scenarios, RKHS could be the best choice when the computation time and prediction accuracy were comprehensively considered.

Except for GYP, the prediction accuracy of TAMs produced by CMLM was consistently higher than that by FarmCPU. In multiple species, FarmCPU outperformed CMLM and other methods by controlling the inflation of *p* values, identifying newly associated SNPs, and overlapping with the reported loci (Liu et al., 2016). CMLM and FarmCPU use different strategies to solve the confounding problem and improve statistical power for the mixed linear model methods (Zhang et al., 2010; Liu et al., 2016), which results in different marker information. Different markers, marker distributions, MAF, and multicollinearity might show the discrepancy in accuracies of the two GWAS methods. Except for EW, moderate and high accuracies were displayed in five models using FarmCPU-derived TAMs for GYP and other traits, which were high enough to make efficient predictions. GS can remarkably accelerate genetic gains by shortening the breeding cycle even if moderate accuracies are achieved (Heffner et al., 2010).

Genome-wide association study is a rapid and effective method for identifying genetic variations in important germplasms. Based on the prior knowledge of the underlying genetic architecture detected by GWAS, the advantage of integrating GWAS with GS was identified in our association panel. Our results showed that subsets of TAMs that treated significant SNP as the fixed effects or random effects could improve the prediction accuracies of GYP and yield-related traits compared with all markers. This was similar to the results of the studies by Liu et al. (2020) and Yuan et al. (2019), who reported that the prediction accuracy of marker trait-associated SNPs was higher than that of all markers or random genome-wide SNPs for maize grain yield, flowering time, and *Fusarium* ear rot resistance. The study by Lozada et al. (2019) proved that wheat yield achieved higher accuracies using three subsets of associated markers that were selected from GWAS in training populations compared with all markers. Compared with GS without marker selection by GWAS, TAMs as the random effects in GS increased the prediction accuracies, regardless of which TAMs were selected from in the full dataset or training set (Cericola et al., 2017; Liu et al., 2019; Ali et al., 2020). In most cases, the prediction accuracy was the highest at 100–5,000 TAMs and then decreased as the number of markers increased for the eight traits. A similar trend was observed in wheat grain yield based on GWAS-derived markers (Lozada et al., 2019). The decreased trend of the prediction accuracy was also found in many cases where evenly distributed SNPs were used and three examples where randomly selected markers were used in rice (Spindel et al., 2015). Higher marker density caused a lower prediction accuracy if significant SNPs were included, but resulted in a higher accuracy if significant SNPs were excluded for simple traits that were controlled by one or several genes with the large effects (Zhang et al., 2019a). The multicollinearity and complexity of GS models for the estimation of GEBVs became severe when an increasing number of markers were used (Ali et al., 2020), which might decrease the prediction accuracy. The smaller number of TAMs that benefited higher accuracies could be helpful to lower the costs of genotyping in GS-assisted breeding. In general, GS based on GWAS results from the full panel set could help to improve the prediction accuracies, although the “inside trading” effects lead to inflated values (Arruda et al., 2016).

In this study, treating one or several significant SNPs as the fixed effects in GS models resulted in higher accuracies in most cases, compared with those with only the random effects, which was in accordance with the trends in accuracy improvement shown in maize, wheat, and rice (Arruda et al., 2016; Spindel et al., 2016; Herter et al., 2019; Odilbekov et al., 2019). The incorporation of large-effect QTL or SNPs as the fixed effects was also a promising strategy to improve the prediction accuracy of GS (Bernardo, 2014; Herter et al., 2019). A slightly decreased accuracy was observed in the fixed model of GBLUP and RKHS for GYP, EW, and HKW. A similar result was also revealed in wheat yield stability using GBLUP (Sehgal et al., 2020). Except for HKW, the genetic architecture of GYP, EW, and yield stability was complex and hard to capture, which was supported by the fact that less robust SNPs with low phenotypic variation were identified. These could lead to the results obtained for these traits.

Integrating information on population structure into fixed models did not improve prediction performance and, in some cases, slightly decreased the accuracies. Similar results were found in the study by Rio et al. (2019); when taking genetic structure into account, the prediction accuracy of maize grain yield, grain moisture, yield index, and male flowering did not improve compared to standard GBLUP. However, Liu et al. (2019) showed that taking three principal components as the fixed effects in the random model could slightly improve the prediction accuracy. In fact, the impact of population structure on GS accuracy depends on many factors such as *a priori* indicators, prediction strategies, allele effects, allele frequencies between groups, the features of traits, and populations (Guo et al., 2014; Liu et al., 2019; Rio et al., 2019). Extended models that consider this information will guarantee high accuracies of GEBV.

The major limitation of incorporating TAMs into GS models depended on the accuracy of GWAS results. Marker selection strategies based on *p* values or marker effects might produce an improper marker set with low accuracies if the GWAS was incorrect (Jeong et al., 2020). GWAS results from the full data set that included the training set and testing sets might produce an overfitted markers set. In real GS-assisted breeding projects, the training set is used to conduct prediction models and predict other breeding populations that only have genotypes. Further investigation is needed in order to validate the application prospect of GS based on prior information from the GWAS results.

Despite these limitations, the combination of GWAS and GS offers an effective means for germplasm screening of traits with low heritability where, for instance, a 1% increase in prediction accuracy could improve genetic gains (Rice and Lipka, 2019). Furthermore, continued enlargement of the association panel by incorporating new fixed effects and high-quality phenotypic data from multi-environment trials is expected to improve the accuracy of GEBV. Besides, the marker information and training population will be used to obtain an optimum breeding design and improve genetic gains through reducing costs. Recently, GMStool is developed to present the best prediction model with the optimal marker set based on GWAS results (Jeong et al., 2020), which provides a useful tool for breeders. As GBS, SNP array technology, and other high-output genotyping strategies arise, the genotyping costs are likely to continue to decrease, whereas the phenotyping costs are usually steady or increasing (Spindel et al., 2015). Therefore, the combination of GWAS and GS will become a cost-effective method for selecting high-yield germplasms in maize and other species.

## Data Availability Statement

The original contributions presented in the study are included in the article/Supplementary Material, further inquiries can be directed to the corresponding author.

## Author Contributions

JM collected phenotypic data, performed GWAS and GS analyses, and wrote the manuscript. YC provided help for the phenotypic measurement. All authors have read and agreed to the published version of the manuscript.

## Conflict of Interest

The authors declare that the research was conducted in the absence of any commercial or financial relationships that could be construed as a potential conflict of interest.
