# Supplementary material for: Genetic Dissection of Grain Yield of Maize and Yield-Related Traits Through Association Mapping and Genomic Prediction
Source: Front Plant Sci. 2021 Jul 15;12:690059. doi: 10.3389/fpls.2021.690059 (PMC8319912; doi:10.3389/fpls.2021.690059)
Supplement: Supplementary file 1 [file Data_Sheet_1.pdf]

Supplementary Material

Supplementary Figures

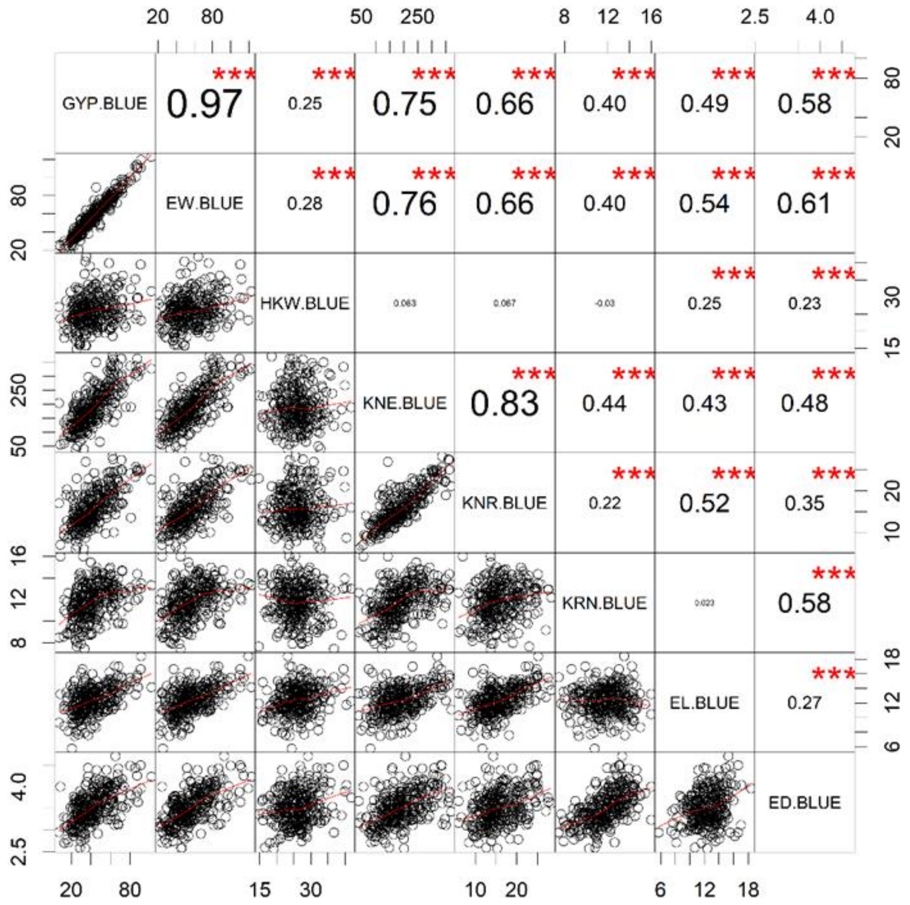

Figure S1 Pairwise correlations between grain yield and yield-related traits based on their best linear unbiased estimate (BLUE) values. The traits of GYP, EW, HKW, KNE, KNR, KRN, EL, and ED are abbreviated from grain yield per plant, ear weight, thousand kernel weight, kernel number per ear, kernel number per row, kernel row number, ear length, and ear diameter, respectively. Numbers with asterisk denote the Pearson's correlation coefficients at different significances (*, 0.05; **, 0.01; ***, 0.001) and the word size of them indicate the correlation level.

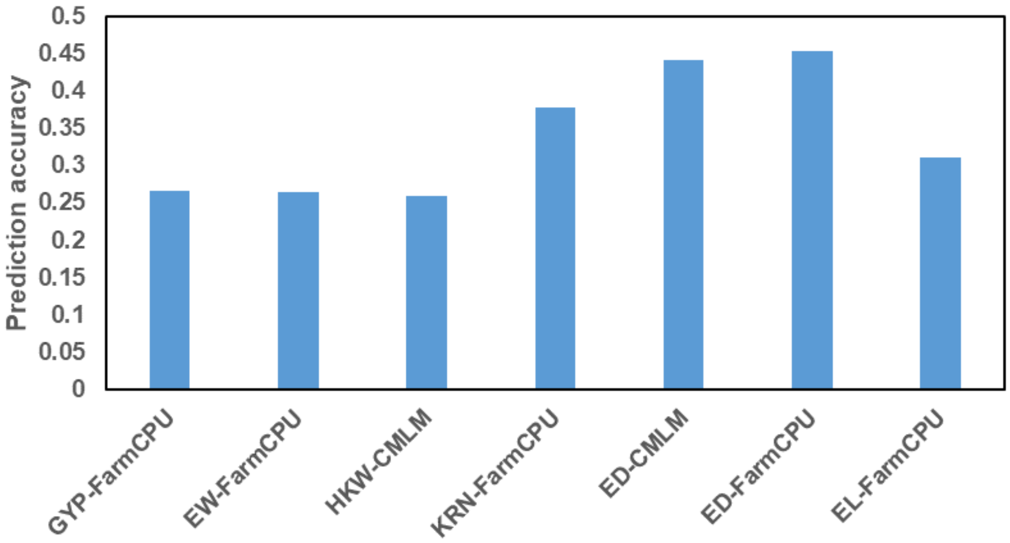

Figure S2 Prediction accuracy of significant SNPs detected using two GWAS methods. The significant SNP information is shown in Table 1. All significant SNPs are treated as random effects in reproducing kernel Hilbert space. GYP, EW, HKW, KRN, EL, and ED are abbreviations of grain yield per plant, ear weight, thousand kernel weight, kernel row number, ear length, and ear diameter, respectively. CMLM, compressed mixed linear model. FarmCPU, fixed and random model circulating probability unification.

Supplementary Tables

Table S1 Phenotypic performance of grain yield and yield-related traits in the association panel

Trait*	Environment§	Mean	Standard error of mean	Coefficient of variation	Skewness	Kurtosis
GYP (g)	YY2017	41.9	1.79	0.65	1.16	1.39
	SY2017	47.49	1.26	0.46	0.65	0.57
	DC2017	39.92	1.56	0.65	1.44	2.27
	YY2019	38.59	1.68	0.65	1.47	3.30
	BLUE	42.2	0.95	0.40	0.71	0.34
EW (g)	YY2017	57.97	2.22	0.58	1.17	1.43
	SY2017	61.15	1.48	0.42	0.59	0.61
	DC2017	57.38	1.97	0.51	1.28	2.50
	YY2019	52.75	1.91	0.54	1.39	2.90
	BLUE	56.9	1.12	0.34	0.68	0.32
HKW (g)	YY2017	29.07	0.38	0.22	1.02	2.61
	YC2017	24.49	0.38	0.25	0.03	-0.26
	SY2017	28.74	0.36	0.22	0.22	0.28
	DC2017	30.18	0.52	0.29	0.50	-0.12
	YY2019	23.46	0.31	0.20	-0.06	-0.19
	BLUE	26	0.26	0.17	0.45	0.68
KNE	YY2017	186.77	6.21	0.50	0.56	0.13
	YC2017	184.02	8.97	0.79	0.85	-0.01
	SY2017	194.45	4.7	0.42	0.31	-0.16
	YY2019	199.23	7.41	0.57	0.64	1.13
	BLUE	189.36	3.91	0.36	0.45	-0.23
KNR	YY2017	15.26	0.39	0.38	5.86	0.56
	YC2017	15.64	0.58	0.57	8.94	0.82
	SY2017	15.38	0.29	0.33	5.04	0.43
	YY2019	18.8	0.4	0.31	5.82	0.95
	BLUE	15.87	0.25	0.28	4.37	0.46
KRN	YY2017	11.8	0.15	0.20	-0.42	0.06
	YC2017	11.8	0.18	0.25	-0.40	-0.45
	SY2017	13.02	0.11	0.15	-0.21	0.16
	YY2019	10.45	0.17	0.28	-0.42	-0.43
	BLUE	11.79	0.09	0.14	-0.21	-0.33
EL (cm)	YY2017	11.74	0.17	0.22	0.22	0.24
	YC2017	13.98	0.17	0.20	0.13	0.70
	SY2017	11.47	0.12	0.18	-0.19	1.00
	DC2017	13.5	0.17	0.21	-0.11	0.57
	YY2019	12.38	0.18	0.23	-0.17	0.34
	BLUE	12.37	0.11	0.15	-0.15	0.27
ED (cm)	YC2017	3.57	0.04	0.21	-0.39	0.27
	SY2017	3.84	0.03	0.12	0.22	0.65

YY2017	3.39	0.04	0.16	-0.38	-0.03
YY2019	3.18	0.04	0.18	-0.34	0.19
DC2017	3.35	0.03	0.17	0.08	-0.08
BLUE	3.52	0.02	0.11	0.28	-0.17

∗ GYP, EW, HKW, KNE, KNR, KRN, EL, and ED are abbreviations of grain yield per plant, ear weight, thousand kernel weight, kernel number per ear, kernel number per row, kernel row number, ear length, and ear diameter, respectively. § The environments of YC, SY, DC, and YY are abbreviated from Yucheng, Sanya, Dancheng, and Yuanyang, respectively; 2017 and 2019 denote years; BLUE, best linear unbiased estimate.

Table S2 Multi-environment analysis of variance and heritability for grain yield and yield-related traits.

Trait[#]	Variance[§]			
	Genotype	Environment	Genotype by environment	Heritability
GYP	266.13**	6.76**	530.02**	0.60
EW	383.66**	7.74**	822.49**	0.59
HKW	17.51**	8.60**	29.67**	0.70
KNE	4269.21**	125.37**	8492.98**	0.61
KNR	16.54**	3.29**	26.66**	0.64
KRN	2.12**	0.94**	3.95**	0.59
EL	3.20**	1.05**	3.09**	0.77
ED	0.13**	0.05**	0.18**	0.73

[#] GYP, EW, HKW, KNE, KNR, KRN, EL, and ED are abbreviations of grain yield per plant, ear weight, thousand kernel weight, kernel number per ear, kernel number per row, kernel row number, ear length, and ear diameter, respectively. [§] **, the effects are significant at 0.01 level.

Table S3 Prediction accuracy of random model based on trait-associated markers in five prediction models for kernel number per ear.

Model[*]	Marker source[§]	Prediction accuracy[#]							
		100[†]	500	1,000	5,000	10,000	20,000	40,000	58,129
Bayes A	CMLM-RAN	0.58 (0.08)	0.62 (0.07)	0.64 (0.07)	0.61 (0.08)	0.58 (0.08)	0.51 (0.09)	0.35 (0.10)	0.16 (0.10)
	FarmCPU-RAN	0.49 (0.09)	0.49 (0.10)	0.50 (0.09)	0.49 (0.09)	0.45 (0.09)	0.40 (0.10)	0.28 (0.10)	
Bayes B	CMLM-RAN	0.55 (0.08)	0.61 (0.08)	0.63 (0.07)	0.59 (0.08)	0.56 (0.08)	0.49 (0.09)	0.31 (0.10)	0.14 (0.10)
	FarmCPU-RAN	0.46 (0.09)	0.47 (0.10)	0.48 (0.09)	0.47 (0.10)	0.44 (0.10)	0.39 (0.10)	0.26 (0.10)	
Bayes C	CMLM-RAN	0.57 (0.08)	0.62 (0.07)	0.64 (0.07)	0.60 (0.08)	0.58 (0.08)	0.51 (0.09)	0.33 (0.10)	0.14 (0.11)
	FarmCPU-RAN	0.48 (0.09)	0.48 (0.10)	0.50 (0.09)	0.49 (0.09)	0.46 (0.10)	0.40 (0.10)	0.27 (0.10)	
GBLUP	CMLM-RAN	0.59 (0.08)	0.64 (0.07)	0.66 (0.07)	0.63 (0.08)	0.60 (0.08)	0.53 (0.09)	0.35 (0.10)	0.15 (0.10)
	FarmCPU-RAN	0.50 (0.09)	0.50 (0.09)	0.52 (0.09)	0.53 (0.09)	0.50 (0.09)	0.44 (0.09)	0.30 (0.10)	
RKHS	CMLM-RAN	0.61 (0.08)	0.66 (0.07)	0.67 (0.07)	0.65 (0.08)	0.62 (0.08)	0.56 (0.09)	0.42 (0.10)	0.31 (0.11)
	FarmCPU-RAN	0.56 (0.08)	0.57 (0.09)	0.58 (0.09)	0.58 (0.08)	0.56 (0.09)	0.52 (0.10)	0.41 (0.10)	

[*] GBLUP, genomic best linear unbiased prediction; RKHS, reproducing kernel Hilbert space. [§] CMLM-RAN and FarmCPU-RAN, traits-associated markers from compressed mixed linear model (CMLM) and fixed and random model circulating probability unification (FarmCPU) are treated as random effects. [†] 100-40,000, number of trait-associated markers. [#] Prediction accuracy is represented by mean and standard deviation in brackets.

Table S4 Prediction accuracy of random model based on trait-associated markers in five prediction models for kernel number per row.

Model*	Scenario§	Prediction accuracy#							
		100†	500	1,000	5,000	10,000	20,000	40,000	58,129
Bayes A	CMLM-RAN	0.56 (0.07)	0.66 (0.07)	0.67 (0.06)	0.66 (0.07)	0.63 (0.07)	0.55 (0.08)	0.39 (0.10)	0.19 (0.10)
	FarmCPU-RAN	0.51 (0.09)	0.53 (0.09)	0.54 (0.08)	0.52 (0.08)	0.50 (0.09)	0.45 (0.09)	0.33 (0.10)	
Bayes B	CMLM-RAN	0.53 (0.07)	0.64 (0.07)	0.65 (0.07)	0.64 (0.07)	0.60 (0.08)	0.53 (0.09)	0.35 (0.10)	0.18 (0.10)
	FarmCPU-RAN	0.49 (0.08)	0.51 (0.09)	0.54 (0.08)	0.51 (0.09)	0.49 (0.09)	0.44 (0.09)	0.30 (0.10)	
Bayes C	CMLM-RAN	0.55 (0.07)	0.66 (0.07)	0.67 (0.06)	0.66 (0.07)	0.63 (0.07)	0.55 (0.08)	0.38 (0.09)	0.18 (0.10)
	FarmCPU-RAN	0.51 (0.08)	0.53 (0.09)	0.54 (0.08)	0.52 (0.08)	0.50 (0.09)	0.45 (0.09)	0.32 (0.10)	
GBLUP	CMLM-RAN	0.58 (0.07)	0.68 (0.06)	0.69 (0.06)	0.68 (0.06)	0.64 (0.07)	0.57 (0.08)	0.39 (0.09)	0.19 (0.10)
	FarmCPU-RAN	0.53 (0.08)	0.56 (0.08)	0.57 (0.08)	0.56 (0.08)	0.54 (0.08)	0.48 (0.09)	0.34 (0.10)	
RKHS	CMLM-RAN	0.56 (0.11)	0.68 (0.11)	0.70 (0.12)	0.68 (0.12)	0.65 (0.11)	0.59 (0.11)	0.46 (0.11)	0.35 (0.11)
	FarmCPU-RAN	0.54 (0.08)	0.61 (0.08)	0.61 (0.07)	0.61 (0.07)	0.60 (0.08)	0.55 (0.08)	0.45 (0.10)	

* GBLUP, genomic best linear unbiased prediction; RKHS, reproducing kernel Hilbert space. § CMLM-RAN and FarmCPU-RAN, traits-associated markers from compressed mixed linear model (CMLM) and fixed and random model circulating probability unification (FarmCPU) are treated as random effects; † 100-40,000, number of trait-associated markers. # Prediction accuracy is represented by mean and standard deviation in brackets.

Table S5 Prediction accuracy of random model, fixed model, and population structure model based on trait-associated markers in five prediction models for ear length.

Model[*]	Scenario[§]	Prediction accuracy[#]							
		100[†]	500	1,000	5,000	10,000	20,000	40,000	58,129
Bayes A	CMLM-RAN	0.68 (0.06)	0.77 (0.05)	0.79 (0.05)	0.78 (0.05)	0.76 (0.06)	0.71 (0.06)	0.56 (0.08)	
	FarmCPU-RAN	0.71 (0.06)	0.65 (0.06)	0.60 (0.06)	0.56 (0.07)	0.54 (0.07)	0.51 (0.08)	0.46 (0.08)	0.40 (0.09)
	FarmCPU-FIX	0.73 (0.06)	0.71 (0.06)	0.70 (0.06)	0.64 (0.06)	0.62 (0.07)	0.59 (0.07)	0.54 (0.08)	
Bayes B	CMLM-RAN	0.67 (0.06)	0.76 (0.05)	0.78 (0.05)	0.77 (0.06)	0.74 (0.06)	0.68 (0.07)	0.54 (0.08)	
	FarmCPU-RAN	0.71 (0.06)	0.68 (0.06)	0.66 (0.07)	0.57 (0.07)	0.55 (0.07)	0.51 (0.08)	0.44 (0.09)	0.39 (0.09)
	FarmCPU-FIX	0.71 (0.06)	0.71 (0.06)	0.71 (0.06)	0.65 (0.06)	0.63 (0.07)	0.59 (0.07)	0.53 (0.08)	
Bayes C	CMLM-RAN	0.68 (0.06)	0.77 (0.05)	0.79 (0.05)	0.78 (0.05)	0.76 (0.06)	0.71 (0.06)	0.56 (0.08)	
	FarmCPU-RAN	0.72 (0.06)	0.67 (0.06)	0.62 (0.06)	0.56 (0.07)	0.54 (0.07)	0.51 (0.08)	0.45 (0.09)	0.40 (0.09)
	FarmCPU-FIX	0.73 (0.06)	0.71 (0.05)	0.70 (0.06)	0.64 (0.07)	0.62 (0.07)	0.59 (0.07)	0.53 (0.08)	
GBLUP	CMLM-RAN	0.69 (0.06)	0.79 (0.05)	0.81 (0.04)	0.79 (0.05)	0.77 (0.06)	0.71 (0.06)	0.56 (0.08)	
	FarmCPU-RAN	0.73 (0.06)	0.66 (0.06)	0.64 (0.06)	0.58 (0.07)	0.56 (0.07)	0.52 (0.07)	0.45 (0.08)	0.40 (0.09)
	FarmCPU-FIX	0.73 (0.06)	0.73 (0.05)	0.73 (0.05)	0.71 (0.05)	0.67 (0.06)	0.60 (0.07)	0.53 (0.08)	
	FarmCPU-FIX-PS	0.73 (0.06)	0.68 (0.06)	0.67 (0.06)	0.64 (0.07)	0.62 (0.07)	0.58 (0.07)	0.53 (0.08)	
RKHS	CMLM-RAN	0.70 (0.05)	0.79 (0.04)	0.81 (0.04)	0.79 (0.05)	0.76 (0.05)	0.70 (0.06)	0.58 (0.08)	
	FarmCPU-RAN	0.73 (0.05)	0.67 (0.06)	0.64 (0.07)	0.61 (0.07)	0.59 (0.07)	0.56 (0.08)	0.51 (0.08)	0.48 (0.09)
	FarmCPU-FIX	0.74 (0.05)	0.69 (0.06)	0.68 (0.06)	0.66 (0.06)	0.64 (0.06)	0.61 (0.07)	0.57 (0.07)	
	FarmCPU-FIX-PS	0.73 (0.06)	0.68 (0.06)	0.67 (0.06)	0.66 (0.06)	0.64 (0.07)	0.61 (0.07)	0.57 (0.08)	

[*] GBLUP, genomic best linear unbiased prediction; RKHS, reproducing kernel Hilbert space. [§] CMLM-RAN and FarmCPU-RAN, traits-associated markers from compressed mixed linear model (CMLM) and fixed and random model circulating probability unification (FarmCPU) are treated as random effects; FarmCPU-FIX, significant SNPs ($P < 1.72E\text{-}05$) are treated as fixed effects and other remaining markers are treated as random effects (fixed model); FarmCPU-FIX-PS, the Q matrix is treated as fixed effect in the fixed model. [†] 100-40,000, number of trait-associated markers. [#] Prediction accuracy is represented by mean and standard deviation in brackets.

Table S6 Prediction accuracy of random model, fixed model, and population structure model based on trait-associated markers in five prediction models for ear diameter.

Model[*]	Scenario[§]	Prediction accuracy[#]							
		100[†]	500	1,000	5,000	10,000	20,000	40,000	58,129
Bayes A	CMLM-RAN	0.71	0.80	0.83	0.83	0.81	0.76	0.60	
		(0.07)	(0.04)	(0.03)	(0.03)	(0.04)	(0.05)	(0.07)	
	FarmCPU-RAN	0.74	0.71	0.67	0.59	0.57	0.54	0.49	
		(0.06)	(0.06)	(0.06)	(0.08)	(0.08)	(0.08)	(0.09)	0.44
	CMLM-FIX	0.71	0.80	0.83	0.83	0.81	0.76	0.65	(0.09)
		(0.06)	(0.04)	(0.04)	(0.04)	(0.04)	(0.05)	(0.07)	
	FarmCPU-FIX	0.75	0.72	0.69	0.63	0.61	0.58	0.53	
		(0.05)	(0.06)	(0.06)	(0.08)	(0.08)	(0.08)	(0.09)	
Bayes B	CMLM-RAN	0.70	0.79	0.82	0.82	0.79	0.73	0.59	
		(0.07)	(0.04)	(0.04)	(0.04)	(0.04)	(0.05)	(0.08)	
	FarmCPU-RAN	0.73	0.71	0.68	0.63	0.59	0.55	0.49	
		(0.06)	(0.06)	(0.06)	(0.07)	(0.08)	(0.08)	(0.09)	0.45
	CMLM-FIX	0.70	0.79	0.82	0.82	0.79	0.74	0.64	(0.10)
		(0.06)	(0.04)	(0.04)	(0.04)	(0.05)	(0.05)	(0.07)	
	FarmCPU-FIX	0.74	0.73	0.71	0.67	0.63	0.59	0.53	
		(0.06)	(0.06)	(0.06)	(0.07)	(0.07)	(0.08)	(0.09)	
Bayes C	CMLM-RAN	0.71	0.80	0.83	0.83	0.81	0.76	0.60	
		(0.07)	(0.04)	(0.03)	(0.03)	(0.04)	(0.05)	(0.07)	
	FarmCPU-RAN	0.74	0.70	0.66	0.59	0.57	0.54	0.49	
		(0.06)	(0.06)	(0.06)	(0.08)	(0.08)	(0.08)	(0.09)	0.44
	CMLM-FIX	0.71	0.80	0.83	0.83	0.81	0.76	0.65	(0.10)
		(0.06)	(0.04)	(0.04)	(0.04)	(0.04)	(0.05)	(0.07)	
	FarmCPU-FIX	0.75	0.71	0.67	0.62	0.61	0.58	0.53	
		(0.06)	(0.06)	(0.07)	(0.08)	(0.08)	(0.08)	(0.09)	
GBLUP	CMLM-RAN	0.72	0.81	0.83	0.83	0.81	0.76	0.61	
		(0.06)	(0.04)	(0.03)	(0.03)	(0.04)	(0.05)	(0.07)	
	FarmCPU-RAN	0.75	0.68	0.64	0.60	0.59	0.56	0.50	
		(0.05)	(0.07)	(0.07)	(0.08)	(0.08)	(0.08)	(0.08)	
	CMLM-FIX	0.71	0.81	0.83	0.83	0.81	0.76	0.65	
		(0.06)	(0.04)	(0.03)	(0.04)	(0.04)	(0.05)	(0.07)	0.46
	CMLM-FIX-PS	0.73	0.81	0.83	0.83	0.80	0.75	0.67	(0.09)
		(0.06)	(0.04)	(0.03)	(0.04)	(0.04)	(0.05)	(0.07)	
	FarmCPU-FIX	0.75	0.68	0.65	0.62	0.60	0.58	0.58	
		(0.05)	(0.07)	(0.07)	(0.08)	(0.08)	(0.08)	(0.08)	
	FarmCPU-FIX-PS	0.75	0.68	0.65	0.62	0.60	0.58	0.53	
		(0.05)	(0.07)	(0.07)	(0.08)	(0.08)	(0.08)	(0.09)	
RKHS	CMLM-RAN	0.73	0.81	0.84	0.84	0.82	0.76	0.65	
		(0.06)	(0.04)	(0.03)	(0.03)	(0.04)	(0.05)	(0.07)	
	FarmCPU-RAN	0.75	0.70	0.67	0.64	0.63	0.61	0.58	
		(0.05)	(0.06)	(0.07)	(0.08)	(0.08)	(0.08)	(0.09)	
	CMLM-FIX	0.73	0.81	0.83	0.83	0.81	0.75	0.67	
		(0.06)	(0.04)	(0.03)	(0.04)	(0.04)	(0.05)	(0.07)	0.56
	CMLM-FIX-PS	0.73	0.81	0.83	0.83	0.80	0.75	0.67	(0.09)
		(0.06)	(0.04)	(0.03)	(0.04)	(0.04)	(0.05)	(0.07)	
	FarmCPU-FIX	0.75	0.70	0.68	0.64	0.64	0.62	0.59	
		(0.05)	(0.06)	(0.07)	(0.07)	(0.07)	(0.08)	(0.08)	
	FarmCPU-FIX-PS	0.75	0.70	0.67	0.64	0.63	0.62	0.59	
		(0.05)	(0.06)	(0.07)	(0.07)	(0.07)	(0.07)	(0.08)	

[*] GBLUP, genomic best linear unbiased prediction; RKHS, reproducing kernel Hilbert space. [§] CMLM-RAN and FarmCPU-RAN, traits-associated markers from compressed mixed linear model (CMLM) and fixed and random model circulating probability unification (FarmCPU) are treated as random effects; CMLM-FIX and FarmCPU-FIX, significant SNPs (P < 1.72E-05) are treated as fixed effects and other remaining markers are treated as random effects (fixed model); CMLM-FIX-PS and FarmCPU-FIX-PS, the Q matrix is treated as fixed effect in the fixed model. [†] 100-40,000, number of

trait-associated markers. # Prediction accuracy is represented by mean and standard deviation in brackets.
